# Combinations of Mutations Sufficient to Alter *Arabidopsis* Leaf Dissection

**DOI:** 10.3390/plants2020230

**Published:** 2013-04-08

**Authors:** Thomas Blein, Véronique Pautot, Patrick Laufs

**Affiliations:** 1 INRA, UMR1318, Institut Jean-Pierre Bourgin, RD10, F-78000 Versailles, France; E-Mails: Thomas.Blein@versailles.inra.fr (T.B.); Veronique.Pautot@versailles.inra.fr (V.P.); 2 AgroParisTech, Institut Jean-Pierre Bourgin, RD10, F-78000 Versailles, France

**Keywords:** leaf, morphogenesis, differentiation, growth, patterning

## Abstract

Leaves show a wide range of shapes that results from the combinatory variations of two main parameters: the relative duration of the morphogenetic phase and the pattern of dissection of the leaf margin. To further understand the mechanisms controlling leaf shape, we have studied the interactions between several loci leading to increased dissection of the Arabidopsis leaf margins. Thus, we have used (i) mutants in which *miR164* regulation of the *CUC2* gene is impaired, (ii) plants overexpressing *miR319*/*miRJAW* that down-regulates multiple *TCP* genes and (iii) plants overexpressing the *STIMPY*/*WOX9* gene. Through the analysis of their effects on leaf shape and *KNOX I* gene expression, we show that these loci act in different pathways. We show, in particular, that they have synergetic effects and that plants combining two or three of these loci show dramatic modifications of their leaf shapes. Finally, we present a working model for the role of these loci during leaf development.

## 1. Introduction

Leaves are the main plant photosynthetic organs. They show a large variety in their sizes and shapes. Regardless of their final shape, leaves are initiated as small, finger-like primordia from groups of undifferentiated cells, the meristem [1,2]. These primordia will either grow out and rapidly differentiate to generate simple leaves, in which the blade forms a unique unit, or go through additional morphogenetic events that allow the formation of lateral structures, thus generating the leaflets of compound leaves. Different leaf shapes can also be seen as the result of different patterns or levels of dissection: large dissection of the blade generate leaflets, less prominent dissections of the leaf or leaflet margins cause lobes or teeth [3].

Recent molecular genetics have revealed a surprising conservation of the regulatory mechanisms acting at different steps of leaf development, from their initiation at the meristem to the formation of leaflets in the case of compound leaves or to the dissection of the leaf or leaflet margins. Class I *KNOX* (*KNOX I*, *KNOTTED 1-LIKE HOMEOBOX*) genes maintain meristematic cells in an undifferentiated state [4] but also prevent precocious differentiation of primordia in most compound leaves to allow the formation of leaflets [5,6,7]. KNOX I proteins act, in part, through the modulation of hormonal pathways. KNOX I proteins promote cytokinin biosynthesis [8,9] to prolong the morphogenetic phase of compound tomato leaves, enabling leaflet formation [10]. Conversely, KNOX I proteins repress gibberellin biosynthesis [8,11] to prevent differentiation. An increased gibberellin signalling reduces leaflet formation in tomato and smoothens the serrated margin of tomato leaflets or the lobed margins of Arabidopsis leaves overexpressing *KNOX I* genes [12,13]. Whereas *KNOX I* genes maintain cells in an undifferentiated state, class II *TCP* genes (*TEOSINTE BRANCHED1*/*CYCLOIDEA*/*PROLIFERATING CELL FACTOR*) promote cell differentiation [14,15]. Five Arabidopsis *TCP* genes and the tomato *TCP LANCEOLATE* gene are targeted by a miRNA, *miR319* or *miRJAW* [16,17]. Expression of *miR319*-resitant *TCP* genes leads to smaller leaves in Arabidopsis and a simplification of the compound tomato leaf. Conversely, inactivation of *TCP* genes, via for instance the overexpression of *miR319*, leads to prolonged growth leading to larger and serrated leaves in Arabidopsis and to super-compound tomato leaves [14,15,16,17,18,19].

Altogether, the opposite effects of the *KNOX I* and *TCP* genes, and the balance between the cytokinin and gibberellin pathways contribute to determine the window during which the leaf primordium remains undifferentiated and hence is able to respond to morphogenetic clues determining leaflet or teeth formation. Similar to local peaks of auxin response triggering primordium formation at the meristem [20,21], an increase in auxin response is also required for the patterning of leaf primordium to form serrations or leaflets [22,23,24,25,26]. Such an increase in auxin response contributes to the repression of *KNOX I* genes in the meristem during leaf primordium formation and in primordia of compound leaves during leaflet formation [22,25]. Proper individualisation of these auxin-mediated new growth axes (leaf primordia, leaflet primordia and developing teeth) require the activity of the *CUC* (*CUP-SHAPED COTYLEDON*) boundary genes [27,28,29,30,31,32]. Some *CUC* genes, like *CUC2* in Arabidopsis or *SlNAM*/*Goblet* in tomato are targeted by a microRNA, *miR164*. Expression of a *miR164*-resistant *CUC2* gene leads to deeply serrated leaf margins [32,33]. All these different regulators are not acting independently each of the other and numerous connections have been reported, generating an intricate regulatory network. For instance, *CUC* genes and auxin interact [23,31,34] and there is a cross talk between *KNOX I* and *CUC* genes [29,30,35].

Here, to investigate the genetic basis of leaf development we analyse the morphogenetic and genetic effects of combining different mutations that lead to an increased leaf dissection. We used *CUC2g-m4* plants, which expresses a *miR164*-resistant *CUC2* gene, the *mir164a-4* mutant in which the *MIR164A* gene controlling *CUC2* expression during leaf development is inactivated [32], the *jaw-D* mutant, in which the overexpression of *miRJAW*/*miR319* down-regulates five *TCP* genes [17] and *stip-D*, which overexpresses *STIMPY*/*WOX9* that maintains cell division and prevents premature differentiation [36]. We showed previously that leaf dissection in these mutants relies for a large part on a functional *CUC2* gene and that the regulation of *CUC2* by *miR164* is still effective [30]. Here, we further investigate the relationship between these pathways and how they control leaf development.

## 2. Results

### 2.1. Leaf Phenotype of Jaw-D, Stip-D, Mir164a-4/CUC2g-m4 Double and Triple Mutants

#### 2.1.1. *Jaw-D Stip-D* Double Mutant Leaf Phenotype

**Figure 1:**
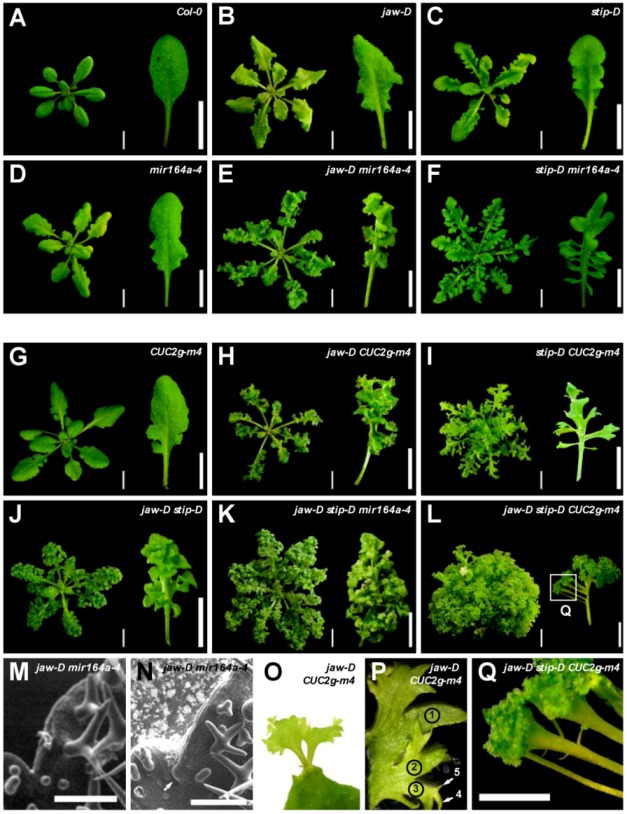
Genetic interactions between *jaw-D*, *stip-D*, *mir164a-4* and *CUC2g-m4*. Rosette and sixth leaf at bolting of the corresponding genotypes. Rosette from (**A**) to (**K**) are bird’s eye views whereas (**L**) is a lateral view of the rosette. (**Q**) is a detail of (**L**). (**M**) and (**N**) show the formation of a tooth of order 2 (arrow) on a *jaw-D mira164a-4* double mutant. (**O**) Sometimes ectopic leaves can be observed in the sinus of *jaw-D CUC2g-m4* double mutants. These ectopic leaves also show the fractal phenotype observed in the main leaves. (**P**) Reiterative growth of the leaf margin: example of the *jaw-D CUC2g-m4* double mutant. The first lobe formed initiates two secondary lobes (label “2”, only one labelled here). This process is reiterated to form the lobes of order 3 to 5 visible in this part of leaf (labels “3” to “5”). Bars = 1 cm in all panels except (**M**) and (**N**) (bars = 100 µm) and (**Q**) (bar = 0.5 cm).

In the *jaw-D* single mutant, the dissection of the leaf margin can be divided in several orders of incision (Figure 1A,B). The first order consists of the main teeth of the leaf (we will call hereafter “teeth” any clear outgrowth at the leaf margin, whatever its size). A second order appears with incisions of the main teeth. Additional orders of incision can occur and reach an order of five, the highest order being observed in the proximal teeth of old leaves. The *stip-D* mutant develops narrow and wavy leaves presenting a single order of dissection (Figure 1C). The *jaw-D stip-D* double mutant has the same order of incision as the *jaw-D* single mutant but the dissections are deeper and can almost reach the midrib in the proximal part of the leaf. In addition, the teeth of this double mutant have a particular form, being narrower in the part close to the midrib and larger towards the outside of the leaf (Figure 1J). 

#### 2.1.2. *Jaw-D Mir164a-4* and *Jaw-D CUC2g-m4* Double Mutant Leaf Phenotype

The *mir164a-4* and *CUC2g-m4* mutants have serrated margins with an order of dissection of 1 or 2 (Figure 1D,G). *jaw-D mir164a-4* [30] and *jaw-D CUC2g-m4* double mutants have curly leaves, with more pronounced dissections (Figure 1E,H). Compared to the single mutants, the dissections of the double mutants are wider and deeper, although they never reach the midrib. The order of dissection is also strongly increased: at bolting it can reach 9 for *jaw-D CUC2g-m4*, and in plants grown for six months in short-day conditions, the order of incision reaches 12 and 15 for the *jaw-D mir164a-4* and *jaw-D CUC2g-m4* double mutants, respectively. The pattern of the different order of teeth is however simple, and can be compared to fractals: on a tooth of a given order, two new teeth appear at the base of the tooth to create a new order (Figure 1M,N). This process is reiterated to give the final leaf shape (Figure 1P). In some cases, ectopic leaves are observed in the sinus (Figure 1O), hinting to the formation of ectopic meristems. 

#### 2.1.3. *Stip-D Mir164a-4* and *Stip-D CUC2g-m4* Double Mutant Leaf Phenotype

The *stip-D mir164a-4* [30] and *stip-D CUC2g-m4* double mutants share a similar type of leaf phenotype characterised by an extreme dissection of the teeth (Figure 1F,I). In *stip-D mir164a-4* the sinus nearly reach the midrib, whereas the dissection is even stronger in the *stip-D CUC2g-m4* double mutant in which the proximal teeth are completely separated one from the other. The constriction at the base of the teeth is also accentuated compared to *stip-D* and *jaw-D stip-D* mutants and in the strongest case the base of the teeth is limited to the central vein of the teeth resembling a petiolule (the petiole-like structure supporting the leaflet). Despite these strong phenotypes, the leaf of these double mutants is still rather flat and not as curled as other double mutants described above.

#### 2.1.4. *jaw-D stip-D mir164a-4* and *jaw-D stip-D CUC2g-m4* Triple Mutant Leaf Phenotype

As the above-described genetic analysis indicates that *stip-D*, *jaw-D* and *mir164a-4*/*CUC2g-m4* control leaf development through different pathways, we constructed the *jaw-D stip-D mir164a-4* and *jaw-D stip-D CUC2g-m4* triple mutants (Figure 1K,L). The *jaw-D stip-D mir164a-4* triple mutant has a leaf phenotype resembling the *jaw-D stip-D* double mutant with a higher order of dissection (Figure 1K). A clear constriction is observed at the base of the teeth of the *jaw-D stip-D mir164a-4* triple mutant. The *jaw-D stip-D CUC2g-m4* triple mutant displays an even stronger leaf phenotype (Figure 1L,Q). The incision of the first proximal teeth of the leaf are so deep that they reach the midrib and that the structure is looking more like leaflets than teeth. In some cases, secondary leaflets with a clear petiolule structure can be observed on the primary petiolule-like structures (Figure 1Q). The lamina is also widely affected, with a higher order of incisions, which gives rise to strong flatness defects. As a result, the leaves look like parsley and the rosette, instead of being flat like in the wild type, adopts a hemispheric shape (Figure 1L).

#### 2.1.5. *Stip-D* Promotes the Formation of Petiole

**Figure 2:**
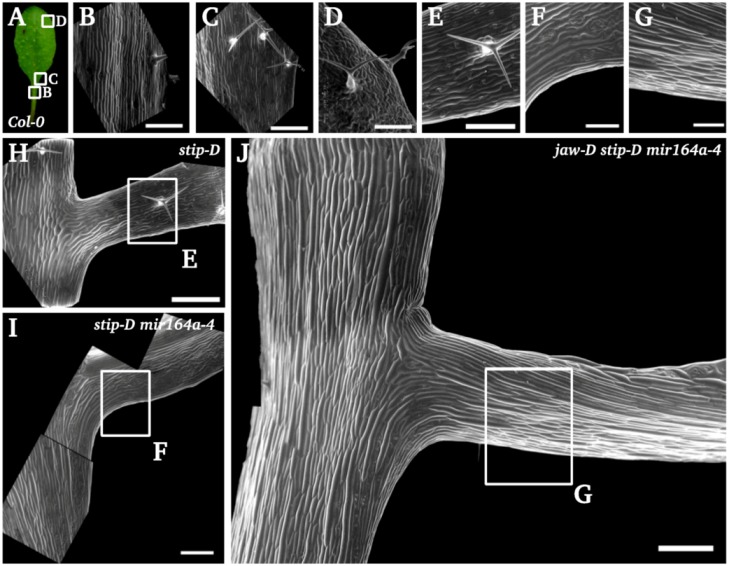
The *stip-D* mutation promotes the formation of petiolule-like structures at the base of the teeth. (**A**–**D**) Epidermal cell structure of the wild-type leaf: The epidermal cells of the petiole are long and narrow, oriented along the proximo-distal axis (**B**). At the junction between the petiole and the lamina some cells are jigsaw-shaped (**C**). On the lamina, three types of cells are observed: jigsaw-shaped cells, stomata cells and long cells along the margin (**D**). (**E**–**J**) The *stip-D* mutation allows the formation of a petiole-like structure at the base of the tooth: In the *stip-D* single mutant (**H** and **E**) and *stip-D mir164a-4* double mutant (**I** and **F**), the base of the first teeth is almost entirely covered by long narrow epidermal cells, but rapidly jigsaw-shaped cells cover the tooth region that forms a lamina-like structure. In the *jaw-D stip-D mir164a-4* triple mutant (**J** and **G**) the region of the tooth with long and narrow cells is longer and forms in this case a real petiole without any lamina. Bars: 500 µm (**B**, **C**, **H**–**I**) 250 µm (**D**–**G**).

The progressive formation of petiole-like structures in *stip-D* and multiple mutants containing *stip-D* suggest that *stip-D* may promote the formation of a petiole. To further test this hypothesis, we examined the shape of the epidermal cells by scanning electronic microscopy (SEM). In the wild type, the petiole is covered by elongated and rectangular cells. Similar elongated cells are present in the lamina above the midrib. Other parts of the lamina are covered by jigsaw-shaped pavement cells and a progressive increase in the complexity of the epidermal shapes is observed from the midrib to lateral domains (Figure 2A–D). In the *stip-D* single mutant, the base of the teeth contains some elongated cells (Figure 2E,H). The region that contained such cells is enlarged in the *stip-D mir164a-4* double mutant and in the *jaw-D stip-D mir164a-4* triple mutant (Figure 2F,G,I,J) suggesting that the base of the teeth have acquired petiole characteristics.

### 2.2. Floral Phenotype of jaw-D, stip-D, mir164a-4/CUC2g-m4 Double and Triple Mutants

**Figure 3:**
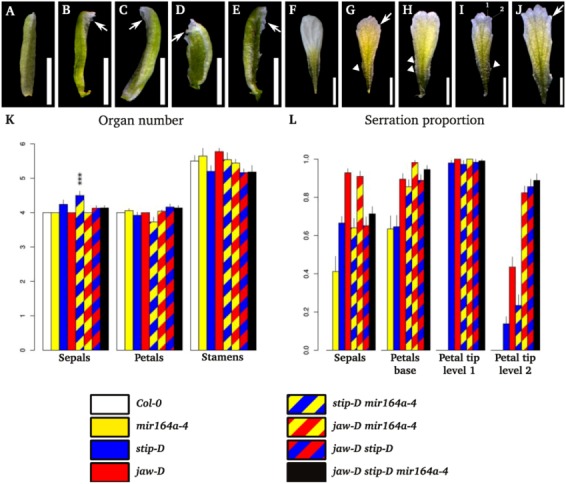
Floral phenotypes. (**A**–**E**) Sepal phenotypes: The wild-type sepal does not exhibit any serration of its margin (**A**). The different mutant combinations show serration of the sepals (arrows) like in *jaw-D* (**B**), *jaw-D mir164a-4* (**C**), *jaw-D stip-D* (**D**), *jaw-D stip-D mir164a-4* (**E**). (**F**–**J**) Petal phenotypes: The wild-type petal does not exhibit any serration of its margin (**F**). The different mutant combinations show serrations of the petals like in *jaw-D* (**G**), *jaw-D mir164a-4* (**H**), *jaw-D stip-D* (**I**), *jaw-D stip-D mir164a-4* (**J**). The serration can occur at the base of the petal (arrow tips) or on the distal end of the petal (arrows). At the distal end, two orders (1 and 2 in **I**) can be distinguished as in leaves. (**K**) Organ number: The number of organs are indicated. 20 flowers from rank 20 to 30 were counted for each genotype. Bars = SE. (**L**) Proportion of serration of the sepals and petals: 20 flowers from rank 20 to 30 were counted for each genotype. Bars = SE.

Floral organs are modified leaves. To investigate if the incision defects observed in leaves are conserved in floral organs, the sepals and petals shapes of the different mutants combinations involving *stip-D*, *jaw-D* and *mir164a-4* were analysed. The numbers of floral organs in the different combinations of mutants varied slightly but these variations are not statistically significant except an increased number of sepals in the *stip-D mir164a-4* double mutant (Figure 3K).

Whereas wild-type sepals have a smooth tip, sepals of the different mutants show serrations at their tips (Figure 3A–E,L). The frequency and level of serration is particularly high in the *jaw-D* single mutant and the *jaw-D mir164a-4* double mutant (Figure 3B,C,L). Additionally, two types of margin serration could be observed in the petals of the different mutants (Figure 3F) compared to the smooth margin of the wild type. First, fine serrations are observed at the base of the petal in all mutant combinations (Figure 3G–J). Second, serrations also form on the distal part of the petal. A first order of incision is present in all mutant combinations involving *jaw-D* or *stip-D*, but not in the single *mir164a-4* mutant. In addition to this first order, a second order of incision can form in mutants carrying the *jaw-D* allele. Almost half of the petals of the *jaw-D* mutant show a second order of incision, whereas almost all petals of double and triple mutants carrying the *jaw-D* mutation show this phenotype (Figure 3L). 

### 2.3. Leaf Phenotype of TCP2 and TCP4 Insertion Lines

**Figure 4:**
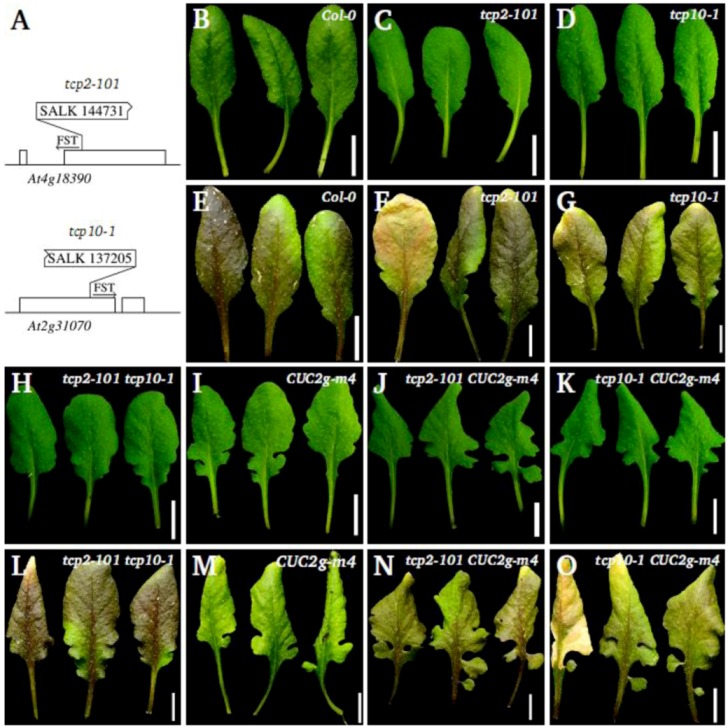
Leaf phenotype of *TCP2* and *TCP10* insertion lines. (**A**) Position of the T-DNA insertion in the *tcp2-101* and *tcp10-1* mutants according to the FST: Rectangles represent the exons. (**B**–**G**) *tcp2-101* and *tcp10-1* single mutants increased their serration when the wild type stopped growing: At bolting, the 6th to 8th leaves of *tcp2-101* (**C**) and *tcp10-1* (**D**) show a slight increase in serration depth compared to wild type (**B**). This difference is accentuated two weeks later (**E**–**G**). H and L The effects of the *tcp2* and *tcp10* mutations are additive: 6th to 8th leaves of *tcp2-101 tcp10-1* double mutants at bolting (**H**) and two weeks later (**L**). I–K, M-O *tcp2-101* and *tcp10-1* increase the serration of the *CUC2g-m4* transgenic mutants: At bolting the 6th to 8th leaves of *tcp2-101 CUC2g-m4* (**J**) and *tcp10-1 CUC2g-m4* (**K**) show an increased serration depth compared to *CUC2g-m4* (**I**). This difference is accentuated two weeks later (**M**–**O**).

The *miR319* miRNA targets five *TCP* genes: *TCP2*, *TCP3*, *TCP4*, *TCP10* and *TCP24* [17]. To get some insight into the contribution of individual *TCP* genes to the phenotype of the *jaw-D* mutant, we analysed the leaf phenotype of two insertion lines in *TCP2* and *TCP10* (Figure 4A). At bolting, *tcp2-101* and *tcp10-1* single mutants exhibit a weak increase of leaf serration, compared to wild type (Figure 4B–D), in agreement with previous observations [19]. This phenotype is more pronounced two weeks after bolting, while the wild-type leaf is not modified during these two weeks (Figure 4E–G). These observations suggest that the down-regulation of any of these two *TCP* genes leads to a prolonged growth period during which the serration of the mutants becomes more pronounced. The *tcp2-101 tcp10-1* double mutant shows a stronger leaf phenotype than the two parental single mutants, with an increased leaf serration already clearly visible at bolting (Figure 4H). Like for the single mutants, this phenotype becomes more pronounced two weeks later (Figure 4L). Furthermore, the distal margins of the teeth are curled downwards.

We next combined either *tcp2-101* or *tcp10-1* with *CUC2g-m4*. The two resulting double mutants show a similar increased leaf serration compared to *CUC2g-m4* single mutant (Figure 4I–K). The serrations are deeper and more teeth present secondary incisions than in the *CUC2g-m4* mutant. Like for the *tcp2-101* and *tcp10-1* single mutants, the phenotype of the double mutants is more pronounced two weeks after bolting whereas the leaf phenotype of the *CUC2g-m4* mutant does not become more pronounced after bolting (Figure 4M–O). In some leaves of the double mutants, the proximal teeth are almost separated from the rest of the lamina.

The *CUC2g-m4* mutant harbours leaves that can not be properly flattened out: in some cases, teeth of the leaf overlap, while the wild-type leaves do not present such phenotype and can easily be flattened out. The *tcp2-101 CUC2g-m4* and *tcp10-1 CUC2g-m4* double mutants also have leaves that could not be properly flattened out, and more overlapping teeth are present compared to the *CUC2g-m4* mutant. A similar phenotype could also be observed in the *jaw-D mir164a-4* and *jaw-D CUC2g-m4* double mutants, though the phenotype was stronger in the combinations with *jaw-D* compared to *tcp2* or *tcp4* mutants (Figure 1E,H). This suggests that the leaf phenotype of these mutants was not only due to an increased incision of the leaf margin but also to an extra growth of the margin compared to wild type.

### 2.4. Expression of KNOX I Reporters

Proper regulation of the *KNOX I* genes is central for leaf development [3,37,38]. Because ectopic expression of *KNOX I* genes in leaves is associated with the formation of increased leaf dissection, we analysed the expression pattern of *KNOX I* genes in different combinations of mutants using GUS reporter constructs for the four Arabidopsis *KNOX I* genes [*KNOTTED-LIKE FROM ARABIDOPSIS THALIANA 1* (*BREVIPEDICELLUS*/*KNAT1*), *KNAT2*, *KNAT6* and *SHOOT MERISTEM LESS* (*STM*)]. 

**Figure 5:**
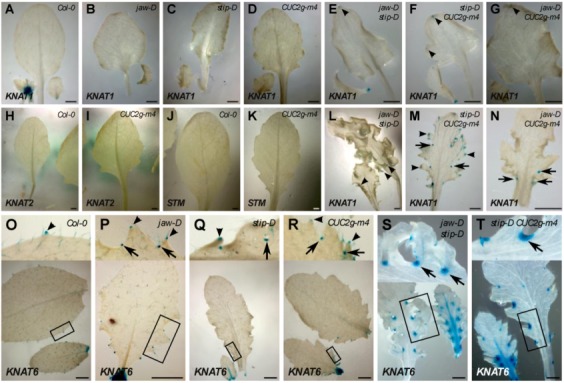
*KNOX I* expression in the leaf of *jaw-D*, *stip-D*, *CUC2g-m4* and different double mutants combinations. (**A**–**G**), (**L**–**N**) *KNAT1:GUS* is expressed in double mutant leaves but not in single mutant leaves: Expression of the *KNAT1:GUS* reporter is absent from leaves of wild type (**A**), *jaw-D* (**B**), *stip-D* (**C**) and *CUC2g-m4* (**D**). In contrast, *KNAT1:GUS* expression can be detected in the the hydathodes (arrowheads) of *jaw-D stip-D* (Eand L), *stip-D CUC2g-m4* (**F** and **M**) and *jaw-D CUC2g-m4* (**G**). In addition, *KNAT1:GUS* is expressed in the distal sinus (arrows) of *stip-D CUC2g-m4* (**M**) and *jaw-D CUC2g-m4* (**N**). (**H**–**K**) *KNAT2* and *STM* genes are not expressed in the leaves of *CUC2g-m4:* No KNAT2*:GUS* expression can be detected in wild type (**H**) and *CUC2g-m4* (I) leaves. No *STM:GUS* expression can be detected in wild type (**J**) and *CUC2g-m4* (**K**) leaves. (**O**–**T**) *KNAT6* expression in *jaw-D*, *stip-D* and *CUC2g-m4* mutants: *KNAT6:GUS* expression is detected in the hydathodes (arrowheads) of all genotypes tested. In *jaw-D*, expression of *KNAT6:GUS* could be detected in the hydathodes of new growing teeth close to the sinus (**P**, arrowheads), but not in the sinus. In *stip-D* (**Q**), *CUC2g-m4* (**R**) *jaw-D stip-D* (**S**) and *stip-D CUC2g-m4* (**T**), *KNAT6:GUS* expression can be detected in the sinus of the teeth (arrows).

The *pKNAT1:GUS* reporter has been shown to be often ectopically expressed in lobed leaves of mutants [6,39,40]. No ectopic *KNAT1* expression is observed in the *jaw-D*, *stip-D* or *CUC2g-m4* single mutants (Table 1 and Figure 5A–D). In contrast, *KNAT1* expression could be observed in the tips of the serrations of *jaw-D stip-D*, *stip-D CUC2g-m4* and *jaw-D CUC2g-m4* double mutants (Figure 5E–G,L,N). In addition, in *jaw-D CUC2g-m4* and *stip-D CUC2g-m4* double mutants, *KNAT1* expression is also observed in the sinus of some teeth (Figure 5M,N).

**Table 1:** Summary of KNOX gene expression patterns in leaves.

	STM:GUS	KNAT1:GUS	KNAT2:GUS	KNAT6:GUS
				tips
WT	N.D.	N.D.	N.D.	
				trichomes
				tips
*CUC2g-m4*	N.D.	N.D.	N.D.	sinus
				trichomes
				tips
*stip-D*	N.D.	N.D.	N.D.	sinus
				trichomes
				tips
*jaw-D*	N.D.	N.D.	N.D.	sinus
				trichomes
		tips		tips
*jaw-D stip-D*	N.D.		N.D.	sinus
				trichomes
		tips		tips
*stip-D CUC2g-m4*	N.D.	sinus	N.D.	sinus
				trichomes
		tips		tips
*jaw-D CUC2g-m4*	sinus	sinus	N.D.	sinus
	petiole	vasculature		trichomes
N.D.: Not Detected				

Expression of the *pSTM:GUS* reporter is observed only in the *jaw-D CUC2g-m4* double mutant and in neither of the other single or double mutants (Table 1 and Figure 5J,K). 

In the wild type, activity of the *pKNAT6:GUS* reporter is observed in the tips of the serrations and at the base of the trichomes (Figure 5O). *pKNAT6:GUS* is similarly expressed in the tips of the serrations and trichomes of the three single mutants. An additional activity of the reporter is detected in the sinus of all mutants (Figure 5P–T). In the double *jaw-D stip-D* and *stip-D CUC2g-m4* the expression in the sinus is very strong (Figure 5S–T).

No ectopic expression of the *pKNAT2:GUS* reporter is observed in any of the mutants analysed (Table 1 and Figure 5H–I).

Altogether, this shows that *CUC2g-m4* is sufficient to induce ectopic expression of *KNAT6* in the sinus. To obtain a similar *KNAT1* ectopic expression, the presence of *jaw-D* combined with either *stip-D* or *CUC2g-m4* is required (Table 1). This revealed a specific effect of *CUC2g-m4* on some members of the Arabidopsis *KNOX I* family and a cooperative effect between the three different mutations analysed here.

## 3. Discussion

Here, we investigated the relationship between different genes regulating leaf dissection and analysed their combined effects on leaf shape and expression of the *KNOX I* genes. First, we show that the abnormal leaf phenotype of *jaw-D*, *stip-D* and *CUC2g-m4* mutants can be further increased when any of these mutations are combined. Second, *jaw-D*, *stip-D* and *CUC2g-m4* have different, and sometimes synergistic effects on *KNOX I* expression on leaves. Altogether, this confirms and extends the previous conclusion [30] that *jaw-D*, *stip-D* and *CUC2g-m4* control leaf development via independent pathways.

**Figure 6:**
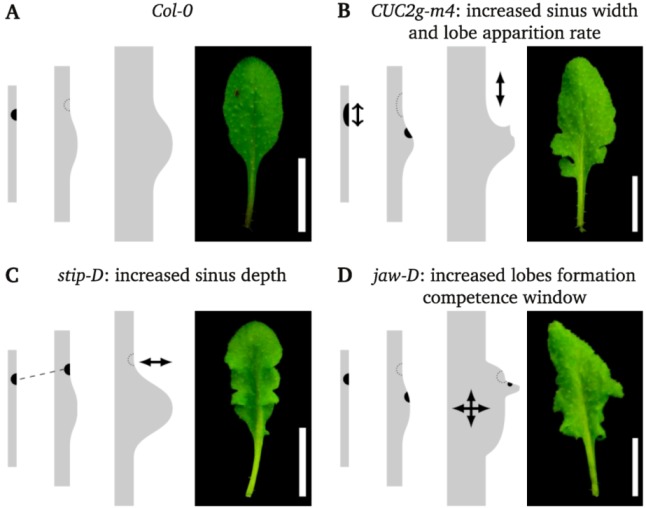
Working model for the effects of *CUC2g-m4*, *stip-D* and *jaw-D* on *CUC2* activity and margin development. The *CUC2* activity (black) and leaf shape at three stages of development are shown for the four genotypes. The expression of *CUC2* in the future distal sinus of the leaf is represented only at a young stage (**A**). Once the primary lobes have formed, the competence to initiate additional lobes ceases. In the *CUC2g-m4* mutant, the expression of *CUC2* is extended in width leading to an enlargement of the sinus. At the same time, *CUC2* expression is reinitiated more rapidly in the primary lobe, leading to a second order of incision and to a secondary lobe (**B**). The competence to form lobes ceases rapidly however limiting the lobe order to two. In the *stip-D* mutant, the *CUC2* expression pattern is the same as in wild type except that *CUC2* expression is prolonged leading to an increased serration depth (**C**). In the *jaw-D* mutant, the duration of the window of competence to form lobes is increased, allowing the reiterative expression of *CUC2* and the formation of several orders of lobes (**D**).

The most striking phenotype of the mutants analysed here is the reiterative dissection process leading to several orders of dissection that dramatically modify leaf shape. On the basis of the leaf shape of the different mutants and combination of mutants, we propose a working model for the effect of the *CUC2g-m4*, *jaw-D* and *stip-D* transgenes on leaf development (Figure 6) that underlines the role of several factors controlling the formation of leaves with complex shapes.

The first factor is the competence to form teeth that seems to be controlled by the *TCP* genes. We have down-regulated the *TCP* genes through two different strategies: first by knocking-out individually or in combination the *TCP2* and *TCP10* genes, and second, via the over-expression of *miR319* in *jaw-D*. In both cases an increased leaf serration was observed, though at different levels. Conversely, expression of *TCP* genes resistant to *miR319* leads to completely smooth leaves [17,18]. Therefore, a window of competence for teeth growth is setup by the action of the *TCP* genes and may be linked to their role in promoting differentiation [14]. This definition of a competence window by the TCP proteins is similar to the one proposed for tomato (*Solanum lycopersicum*). In tomato, the down-regulation of *TCP* genes through the over-expression of *miR319* leads to leaflets of higher order. Conversely, a *miR319*-resistant form of the *LANCEOLATE* (*LA*) *TCP* gene leads to simple tomato leaves [16]. Therefore, the *TCP* genes may control in both Arabidopsis and tomato a window of competence to form leaf sub-units (either teeth or leaflets, Figure 6A,D). 

The second factor is the patterning action of the *CUC2* gene to instruct the formation of serrations. Formation of the serrations may involve local growth inhibition where *CUC2* is expressed, in agreement with the role of *CUC* genes during organ primordium formation [41], but *CUC2* may also have a promoting effect on teeth growth similar to the role of the *NAM*/*CUC* genes during leaflet growth [29]. Indeed, based on the comparison of wild-type and *cuc2* mutant leaf outlines, Kawamura *et al.*, suggested that *CUC2* promotes teeth outgrowth [31]. In *CUC2g-m4*, expression of *CUC2* is enlarged leading to wider and deeper sinus. Teeth of *CUC2g-m4* leaves overlap, whereas wild-type leaves could be mostly flattened out without any overlapping teeth. This also supports the role of *CUC2* in the promotion of teeth outgrowth. 

Large teeth of *CUC2g-m4* also tend to harbour a second order of small teeth at the base of the first order teeth, which is not observed in the wild type. One possible explanation for this would be that formation of higher order of dissections would be controlled by a spacing mechanism such as lateral inhibition. Tips of the first order of serration would locally inhibit the formation of secondary serrations. As it has been shown that auxin distribution underlines first order teeth patterning [23], it is possible that tips of developing serrations deplete auxin from their surroundings, thus inhibiting the initiation of teeth of higher order. The base of the larger teeth of *CUC2g-m4* would be outside the zone from which auxin is depleted and able to reinitiate the process of teeth formation. In this view, the increase in the order of dissection in the *CUC2g-m4* mutant would be an indirect consequence of the deeper serration. However, in the *stip-D* mutant an increase of the serration is also observed but there is no increase in the order of incision. This is also the case for the *jaw-D stip-D* double mutant, which has the same order of incision as the *jaw-D* mutant despite a much stronger leaf dissection. Therefore, the teeth of higher order in *CUC2g-m4* may reflect a shortening of the range of lateral inhibition, due for instance to modified production, distribution or sensitivity to auxin (Figure 6B). A first step to test these hypotheses will be to perform a precise morphometric analysis of leaf development of these mutants to determine the geometry of the leaf margin at the time when the different orders of serrations are initiated.

When the *CUC2g-m4* construct is combined with *jaw-D*, or with the *tcp2-101* or *tcp10-1* mutants not only is the growth of the serration increased, but also their dissection, as the teeth are more individualised than in any of the parental mutants. This is difficult to reconcile with *jaw-D* only controlling teeth growth, and suggests a synergistic effect between both constructs. Koyama *et al.*, showed that TCP proteins activate the expression of *MIR164A*, a negative regulator of *CUC2* [42]. This can, however, not account for the synergistic effect between *jaw-D* and *CUC2g-m4*, as *CUC2g-m4* is insensitive to *miR164*. The same group showed previously that the expression of a chimeric repressor form of *TCP3* (*TCP3SRDX*), leads to the ectopic expression of *CUC3* that is not a target of *miR164*. TCP proteins may therefore have an additional effect on *CUC* gene expression independent of *miR164* [18]. Finally, because a snapdragon CUC orthologue CUPULIFORMIS (CUP) interacts with a TCP protein in a two-hybrid test [43], interactions between CUC2 and TCP proteins may also contribute to the regulation of their activities.

In the *stip-D* mutant, and all mutant combinations including *stip-D*, a strong increase of the depth of the dissection between the teeth is observed. This phenotype is increased when the regulation of *CUC2* by *miR164* is disabled, suggesting another synergistic effect between *CUC2* and *stip-D*. As *stip-D* increases the depth of the dissection between teeth, it could indicate that *stip-D* prolongs the expression of *CUC2* (Figure 6C). This hypothesis is consistent with the recent finding that two genes related to *STIMPY* are positive regulators of *CUC2* during embryo development [44].

## 4. Experimental Section

### 4.1. Plant Material

All material is in Columbia background. The *jaw-D* [17], *stip-D* [36], *mir164a-4*, *CUC2g-m4* [32], *pSTM:GUS* [45], *pKNAT1:GUS* [46], *pKNAT2:GUS* [46], *pKNAT6:GUS* [47]. The *tcp2-101* mutant is the Salk_144731 line [48] and the *tcp10-1* mutant is the Salk_137205 previously described [42]. Plants were grown in long days in greenhouses or in short days in a controlled environment. The controlled long-day growth conditions consisted of 16 h of light at 23 °C and 8 h of darkness at 15 °C. The short-day growth conditions consisted of 8 h light at 23 °C and 16 h darkness at 18 °C. The following primers were used to genotype TCP2 (TCP2-Fwd and TCP2-Rev), tcp2 (TCP2-Fwd and LBA1), TCP10 (TCP10-Fwd and TCP10-Rev) and tcp10 (TCP10-Rev and LBA1): TCP2-Fwd ACG AAG CTG TAT CTA CTG AC, TCP2-Rev AAA GAT TTC CAA AGA CCC TC, LBA1 TGG TTC ACG TAG TGG GCC ATC G, TCP10-Fwd AAC TTC TGC TAT CCT TTC CA, TCP10-Rev ATC CCA TGA GAA CCA TAC TG.

### 4.2. GUS Assay

The GUS staining of shoots harvested from plants grown in long day conditions in greenhouses was carried out as described [49], in the presence of 0.5 mM potassium ferri/ferrocyanide. After an over-night staining, the reaction is stopped by a 70% ethanol solution.

### 4.3. Scanning Electron Microscopy

Scanning electron microscopy was carried out as previously described [50]. The different images obtained where then assembled using Hugin software [51]. 

## 5. Conclusions

Our work reveals the strong flexibility of leaf development, as it is sufficient to modify the expression of only a few genes to generate extremely diverse leaf shapes. We have proposed a working model of the effects of the three transgenes analysed here that may help to understand how leaf shape can be modulated. Validation of this model awaits however tracking-down at their origin the changes in leaf shape that we describe here essentially for mature leaves and linking the dynamic changes in leaf geometry with changes in gene activity. 
